# Myocardial Infarction With Non-obstructive Coronary Arteries in a Nigerian Middle-Aged Woman: A Case Report

**DOI:** 10.7759/cureus.89511

**Published:** 2025-08-06

**Authors:** Olurotimi J Badero, Bamikole Osibowale, Francis C Asogwa, Adebomi Dosumu, Olutomiwa Omokore

**Affiliations:** 1 Department of Interventional Cardiology, Iwosan Lagoon Hospitals, Lagos, NGA; 2 Department of Interventional Cardiology, Division of Cardio-Nephrology, Cardiac Renal & Vascular Associates, Jackson, USA; 3 Department of Cardiology, Caribbean Tristate Heart Institute, Port of Spain, TTO; 4 Department of Internal Medicine, Iwosan Lagoon Hospitals, Lagos, NGA; 5 Department of General Medicine, Iwosan Lagoon Hospitals, Lagos, NGA; 6 Department of Internal Medicine, Babcock University Teaching Hospital, Ilishan-Remo, NGA; 7 Department of Internal Medicine, Benjamin S. Carson College of Health and Medical Sciences, Ilishan-Remo, NGA

**Keywords:** acute myocardial infarction, cardiac arrest, coronary artery disease, coronary microvascular dysfunction, myocardial infarction with non-obstructive coronary arteries

## Abstract

Myocardial infarction with non-obstructive coronary arteries (MINOCA) is a group of heterogeneous diseases with different pathological mechanisms. It is often under-recognized because of its diverse differential diagnoses like myocarditis, takotsubo cardiomyopathy, spontaneous coronary artery dissection (SCAD), coronary microvascular dysfunction, vasospasm, coronary erosion, and embolism. Evaluation with multimodality imaging including intravascular coronary imaging and cardiac magnetic resonance is often necessary to determine the underlying etiology and management.

We report a case of MINOCA in Nigeria involving a 55-year-old woman with metabolic syndrome who experienced cardiac arrest following an intra-abdominal procedure with an electrocardiogram (ECG) and biomarkers consistent with myocardial injury but had non-obstructive coronary artery disease (CAD) on angiography. This case underscores the need to consider MINOCA in patients presenting with myocardial infarction and non-obstructive coronary angiogram, particularly those with multiple comorbidities, and the need for a comprehensive diagnostic approach to uncover the underlying mechanism. Our report encourages increased clinical index of suspicion of this condition while contributing to the growing body of knowledge of this underdiagnosed clinical entity.

## Introduction

The majority of acute myocardial infarctions (MI) are attributed to the rupture or erosion of atherosclerotic plaques, leading to a supply-demand mismatch due to a significant coronary artery stenosis [1]. However, a distinct clinical syndrome known as myocardial infarction with non-obstructive coronary arteries (MINOCA) is characterized by clinical evidence of MI in the presence of normal or near-normal coronary arteries on angiography (defined as stenosis severity <50%) [2]. It is characterized by acute MI based on serial rise or fall in cardiac troponin, symptoms of myocardial ischemia, ischemic changes or pathologic Q waves on electrocardiogram (ECG), loss of viable myocardium or wall motion abnormalities on imaging, coronary thrombus identified on angiography, or autopsy with an absence of obstruction in any major epicardial artery based on angiography, absence of clinically overt alternative for acute presentation, and absence of non-ischemic causes of myocardial injury [3].

Epidemiological studies have indicated that MINOCA is not uncommon, with a reported prevalence ranging from 1% to 15% among patients presenting with acute MI and a mean prevalence of approximately 6% [4,5]. A meta-analysis of 23 studies estimated the prevalence of MINOCA to be 8.1% in a large cohort of over 800,000 consecutive acute MI patients [6]. The worldwide incidence of MINOCA varies between 2.9% and 10.2%. MINOCA is not innocuous, and its mortality pattern is similar to those observed in obstructive MI [7]. Management of MINOCA is conservative with close in-patient monitoring, low-dose aspirin, and statins; calcium channel blockers, renin-angiotensin system blockers, and beta blockers may also be beneficial in some situations [8].

We report a case of MINOCA in a Nigerian middle-aged woman diagnosed angiographically. Our diagnosis of MINOCA in this case is premised on ST-segment elevation on ECG, elevated cardiac injury biomarker, and non-obstructive coronary angiogram.

## Case presentation

A 55-year-old obese woman with a past medical history significant for hypertension and type 2 diabetes mellitus was admitted to our hospital for an endoscopic retrograde cholangiopancreatography (ERCP) and biliary stenting due to pancreatic cancer.

Shortly after the procedure, the patient suffered two episodes of cardiac arrest requiring successful resuscitation before being transferred to the intensive care unit (ICU).

On arrival in the ICU, within an hour after the successful resuscitation, physical examination revealed a dehydrated, afebrile female in respiratory distress (respiratory rate of 40 cycles/min). She was hypoxic (oxygen saturation was 88% on room air) with no signs of anemia or peripheral edema and tachycardic with a pulse rate of 142 beats/min and an elevated blood pressure of 191/125 mmHg. Cardiovascular examination revealed normal S1 and S2 heart sounds with no audible murmurs. She was promptly intubated and placed on mechanical ventilatory support.

The results of investigations are shown in Table 1.

**Table 1:** Results of laboratory investigations INR: international normalized ratio

Test	Value	Reference range
Troponin I	0.15 ng/ml (initial), 0.39 ng/ml (6 hours later)	0-0.04 ng/ml
Creatine kinase	22.5 U/L	22-198 U/L
Blood count		
White blood cell count	23,000/µL	4,000-11,000/µL
Neutrophils	81%	40-70%
Platelets	294,000/µL	150,000-400,000/µL
Hemoglobin	11.2 g/dL	12-16 g/dL
C-reactive protein	3.59 mg/L	0-10 mg/L
D-dimer	>3,000 ng/ml	<500 ng/ml
Renal function test		
Urea	64 mg/dL	5-25 mg/dL
Creatinine	1.4 mg/dL	0.6-1.1 mg/dL
Clotting profile		
Prothrombin time	15.7 seconds	11-13.5 seconds
INR	1.39	0.8-1.1
Liver function test		
Alkaline phosphatase	260 IU/L	45-125 IU/L
Aspartate aminotransferase	26 IU/L	0-40 IU/L
Gamma-glutamyl transferase	784 IU/L	8-54 IU/L
Alanine aminotransferase	25 IU/L	0-40 IU/L
Total bilirubin	2.5 mg/dL	0.2-1.2 mg/dL
Direct bilirubin	2.1 mg/dL	0.0-0.3 mg/dL
Lipid profile		
Total cholesterol	112 mg/dL	<200 mg/dL
High-density lipoprotein	45 mg/dL	>60 mg/dL
Low-density lipoprotein	48.2 mg/dL	<100 mg/dL
Triglyceride	94 mg/dL	<150 mg/dL
Arterial blood gas		
pH	7.11	7.35-7.45
Partial pressure of carbon dioxide	25 mmHg	33-44 mmHg
Partial pressure of oxygen	32 mmHg	75-105 mmHg
Bicarbonate	8.0 mmol/L	22-29 mmol/L
Lactate	0.81 mmol/L	0.5-1.0 mmol/L

**Figure 1:**
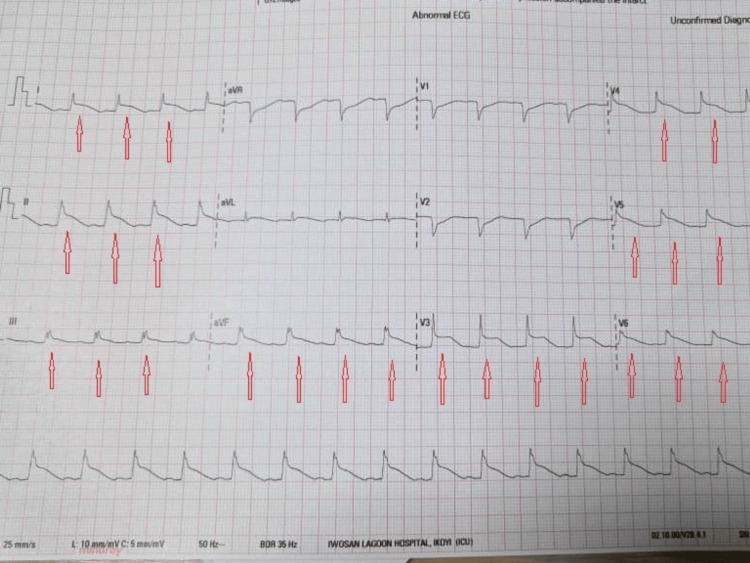
ECG showing sinus tachycardia and inferior and anterolateral ST-segment elevation ECG: electrocardiogram; aVR: aortic valve replacement; aVL: augmented vector left; aVF: augmented vector foot

ECG showed sinus tachycardia, septal Q wave, and inferior and anterolateral ST-segment elevation (Figure 1).

A chest radiograph revealed no evidence of acute cardiopulmonary pathology. Echocardiography demonstrated thickened posterior and lateral walls with left ventricular (LV) concentric hypertrophy and a good LV ejection fraction >55%. There was no significant valvular regurgitation or stenosis.

**Figure 2:**
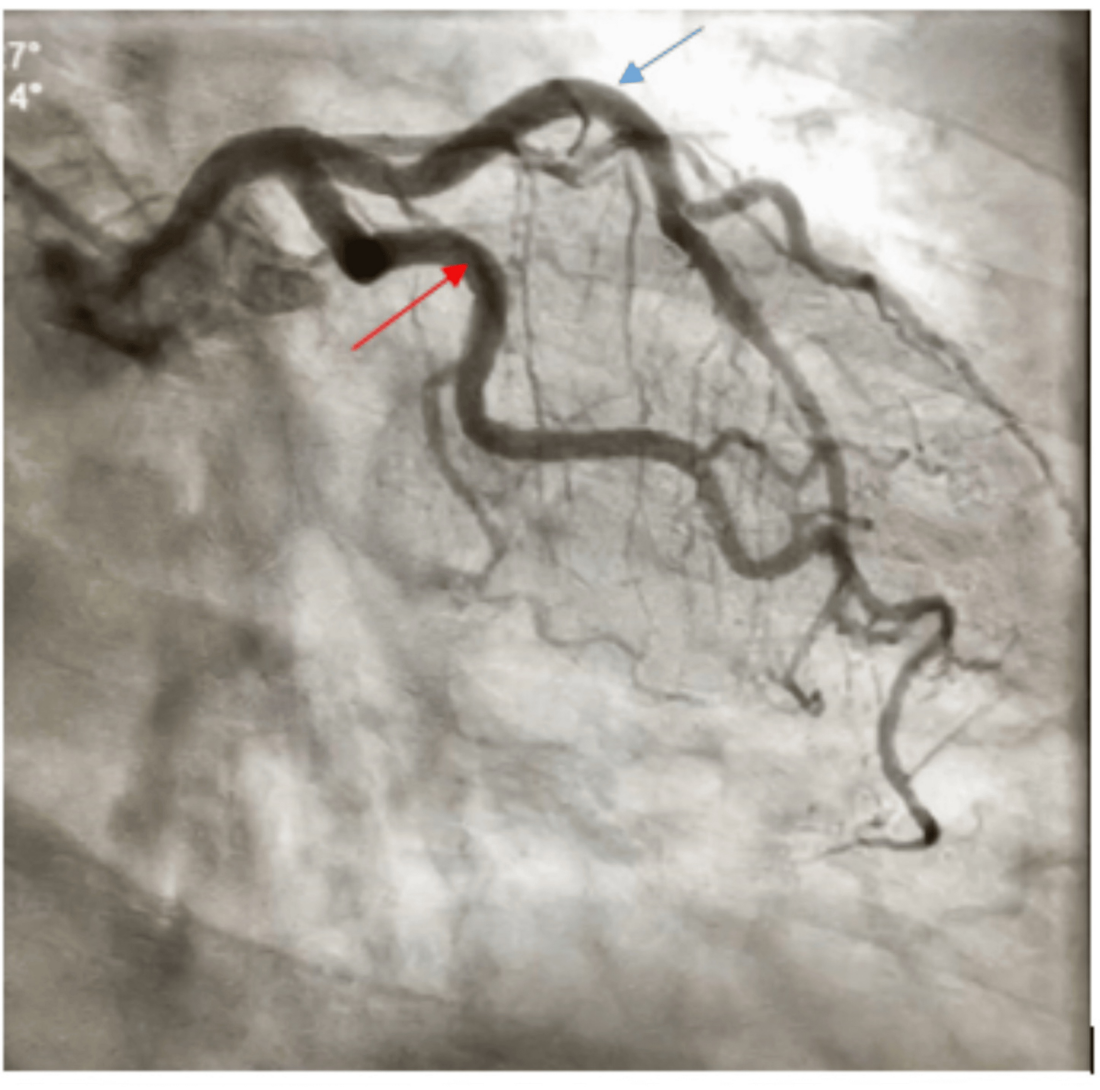
Coronary angiogram showing normal left anterior descending artery (blue arrow) and left circumflex artery (red arrow)

**Figure 3:**
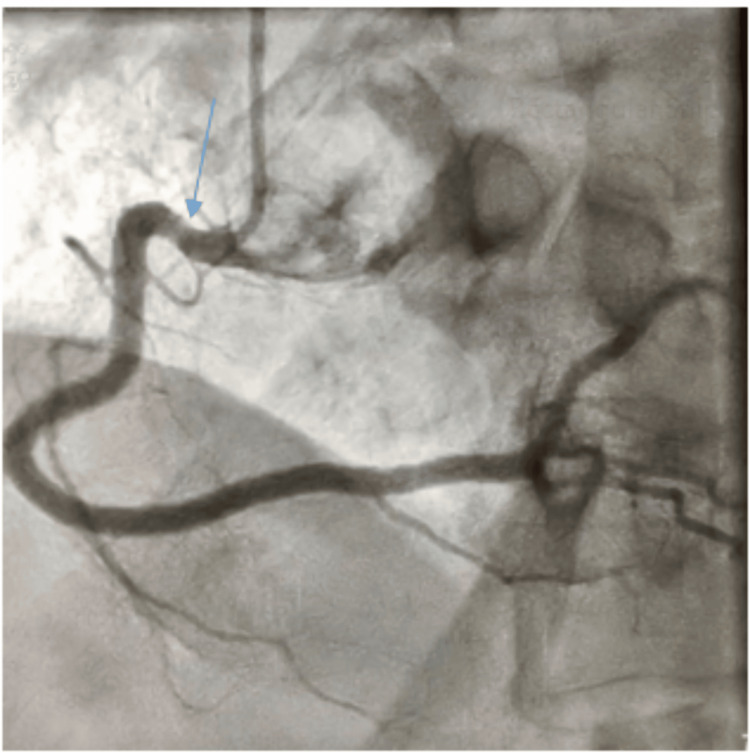
Coronary angiogram showing non-significant stenosis of the proximal right coronary artery (blue arrow)

A computed tomography pulmonary angiography excluded pulmonary embolism. The serial cardiac enzyme (troponin I) six hours later was elevated at 0.39 ng/ml (normal range: 0-0.04 ng/ml). Based on the clinical presentation, ECG findings, and rising troponin, a diagnosis of cardiac arrest secondary to ST-segment elevation myocardial infarction (STEMI) was made. The patient was administered aspirin 300 mg via nasogastric tube, commenced on anticoagulation with subcutaneous enoxaparin at 80 mg daily, and was placed on 100% oxygen. Arrangements were made for urgent coronary angiography. She was subsequently transferred to another facility for this procedure, eight hours after the initial arrival at the ICU. Coronary angiography at the receiving facility revealed non-obstructive coronary arteries (Figure 2 and Figure 3).

Anticoagulation and antiplatelet were continued, but she passed away the next day within 24 hours post-ERCP while a cardiac MRI was being planned while on supportive care.

## Discussion

MINOCA has been recognized as a clinical entity for decades but was first introduced in the Fourth Universal Definition of Myocardial Infarction (UDMI), a consensus statement by the American Heart Association (AHA), American College of Cardiology (ACC), European Society of Cardiology (ESC), and World Heart Federation (WHF) in 2018 [9]. It represents a heterogeneous group of disorders that meet the diagnostic criteria of MI in the absence of significant coronary obstruction with variable underlying pathophysiological mechanisms that include coronary plaque rupture, ulceration, fissuring, vasospasm, embolism, and endothelial dysfunction among others [10]. The pathophysiological mechanisms underlying MINOCA are broad and are classified as atherosclerotic and non-atherosclerotic mechanisms [11]. The atherosclerotic causes are characterized by either a plaque rupture with a break in the fibrous cap, a plaque erosion where thrombus forms on an intact fibrous cap, or calcific nodules. Plaque disruption usually initiates a cascade of events including thrombosis, MI, distal embolization, eventual thrombolysis, and coronary artery spasm presenting as MINOCA [12]. The non-atherosclerotic mechanisms usually involve coronary microvascular dysfunction, coronary embolism, and vasospasm [8]. Increased vascular tone, coronary micro-embolization, and dysregulated nitric oxide production are some of the proposed mechanisms for microvascular dysfunction [13].

The diagnosis of MINOCA warrants further investigation to identify the pathophysiological mechanism and prognosticate, risk-stratify, and institute a definitive treatment strategy. While the ECG and cardiac biomarkers in this patient indicated myocardial injury, further investigations such as coronary intravascular ultrasound (IVUS), optical coherence tomography (OCT), and cardiac magnetic resonance imaging (CMRI) would have provided valuable insights into the underlying etiology of MINOCA in this patient. However, these imaging modalities to evaluate intravascular coronary anatomy as well as the myocardium were not readily available constituting a diagnostic limitation typically seen in resource-poor settings. These modalities have been shown to be helpful in uncovering the mechanisms driving MINOCA and guiding therapeutic interventions, as both diagnostic and therapeutic challenges persist in this entity.

The traffic light sequence, a clinical diagnostic algorithm for MINOCA, begins with the red section, which involves an evaluation to exclude non-ischemic causes of myocardial injury. The evaluation then proceeds to the yellow section, where clinicians consider missed obstructive lesions on the angiogram and assess for subtle non-ischemic mechanisms of cardiac injury using an echocardiogram, angiogram, and CMRI. Although CMRI is a recognized and important investigation, it was not recommended as an essential step in the diagnosis of MINOCA due to its limited availability. After excluding alternative diagnoses, the evaluation moves to the green section, where a diagnosis of MINOCA or CMRI confirmed can be made. Furthermore, in specialized centers, intravascular imaging is available to further elucidate the underlying mechanism [14]. Reynolds et al. [15] in a study evaluating 301 women with MINOCA like our index patient found the etiology in 85% of their cohorts using OCT and CMRI. CMRI is increasingly being recognized as a crucial tool in the evaluation of MINOCA. CMRI can help confirm MI and is also useful in excluding other causes of myocardial injury such as myocarditis, in which there is subepicardial late gadolinium enhancement on the delayed images, and takotsubo cardiomyopathy, in which there are hyperenhancement of the T2-weighted images and lack of late gadolinium enhancement on the delayed images [16]. In our patient, though we could not obtain a CMRI, the ECG and echocardiogram features were not typical of myocarditis or takotsubo cardiomyopathy.

ERCP carries some cardiovascular risks, and there are previous reports of MINOCA following ERCP [17,18]. Cardiovascular complications account for up to 16% of all ERCP-related adverse effects. Chawla and Willingham reported an incidence of 0.07-2.4% in a large prospective study; however, there is a large variation in the incidence in several other studies, mainly due to lack of consensus definition and inconsistent documentation of ERCP-associated cardiovascular complications owing to the transient nature of most of them. Transient complications include desaturation, arrhythmias, and blood pressure irregularities. Significant cardiac complications are due to the interplay of neuroendocrine and mechanical factors; these complications are usually potentiated by the patient's comorbidities and sedation. Conversely, sedation with propofol for therapeutic procedures is associated with procedural success and less cardiovascular complications [19].

The cardiovascular risks following ERCP are particularly higher in those with previous acute coronary syndrome (ACS) and are associated with longer procedure duration, anesthetic medications, background cholangitis, and need for therapeutic intervention [17].

Probable mechanisms in our patient include coronary artery spasm due to procedural stress, elaboration of catecholamine, hypoxia, or coronary thromboembolism which could have resolved spontaneously. Li et al. [20] postulated coronary artery spasm as a probable mechanism in a patient with MINOCA after ERCP.

Previous studies have suggested that patients experiencing major adverse cardiac events following MI tend to have obstructive coronary arteries compared to those with non-obstructive coronary arteries [21]. However, this case highlights that even patients with MINOCA can suffer significant adverse events, as evidenced by the multiple cardiac arrests and eventual death in this patient.

Furthermore, while some research indicated that MINOCA patients tend to have lower mortality rates, better LV ejection fractions, and lower concentrations of cardiac biomarkers compared to those with obstructive coronary artery disease (CAD), others suggested that overall outcomes are similar [22]. 

While more insight is expected to come from the ongoing randomized evaluation of beta blocker and angiotensin-converting enzyme-inhibitor/angiotensin receptor blocker treatment in patients with myocardial infarction with non-obstructive coronary arteries (MINOCA-BAT) trial, the current treatment strategy for MINOCA relies on the results from observational studies which suggest better outcomes with the use of antiplatelet drugs, statins, renin-angiotensin system blockers, and beta blockers [23].

## Conclusions

Ultimately, MINOCA remains a working diagnosis requiring further investigations that are sometimes not readily available in resource-limited settings like ours. It remains an important consideration in patients with MINOCA. Further research is essential to gain a more comprehensive understanding of the diverse underlying etiologies of MINOCA and to develop evidence-based guidelines for its management, from initial presentation to treatment strategies.
